# Cell Death in Hepatocellular Carcinoma: Pathogenesis and Therapeutic Opportunities

**DOI:** 10.3390/cancers14010048

**Published:** 2021-12-23

**Authors:** Ester García-Pras, Anabel Fernández-Iglesias, Jordi Gracia-Sancho, Sofía Pérez-del-Pulgar

**Affiliations:** 1 Liver Unit, Hospital Clínic, University of Barcelona, IDIBAPS, CIBERHED, 08036 Barcelona, Spain; egpras@clinic.cat; 2 Liver Vascular Biology Research Group, IDIBAPS, CIBEREHD, 08036 Barcelona, Spain; afernandezi@clinic.cat; 3 Department for Biomedical Research, Hepatology, Inselspital, University of Bern, 3008 Bern, Switzerland

**Keywords:** programmed cell death, tumor microenvironment, stromal component, nonparenchymal cells

## Abstract

**Simple Summary:**

The progression of liver tumors is highly influenced by the interactions between cancer cells and the surrounding environment, and, consequently, can determine whether the primary tumor regresses, metastasizes, or establishes micrometastases. In the context of liver cancer, cell death is a double-edged sword. On one hand, cell death promotes inflammation, fibrosis, and angiogenesis, which are tightly orchestrated by a variety of resident and infiltrating host cells. On the other hand, targeting cell death in advanced hepatocellular carcinoma could represent an attractive therapeutic approach for limiting tumor growth. Further studies are needed to investigate therapeutic strategies combining current chemotherapies with novel drugs targeting either cell death or the tumor microenvironment.

**Abstract:**

Hepatocellular carcinoma (HCC) is the most prevalent primary liver cancer and the third leading cause of cancer death worldwide. Closely associated with liver inflammation and fibrosis, hepatocyte cell death is a common trigger for acute and chronic liver disease arising from different etiologies, including viral hepatitis, alcohol abuse, and fatty liver. In this review, we discuss the contribution of different types of cell death, including apoptosis, necroptosis, pyroptosis, or autophagy, to the progression of liver disease and the development of HCC. Interestingly, inflammasomes have recently emerged as pivotal innate sensors with a highly pathogenic role in various liver diseases. In this regard, an increased inflammatory response would act as a key element promoting a pro-oncogenic microenvironment that may result not only in tumor growth, but also in the formation of a premetastatic niche. Importantly, nonparenchymal hepatic cells, such as liver sinusoidal endothelial cells, hepatic stellate cells, and hepatic macrophages, play an important role in establishing the tumor microenvironment, stimulating tumorigenesis by paracrine communication through cytokines and/or angiocrine factors. Finally, we update the potential therapeutic options to inhibit tumorigenesis, and we propose different mechanisms to consider in the tumor microenvironment field for HCC resolution.

## 1. Introduction

The liver is an essential organ that exerts important and critical functions (glucose storage, lipid and cholesterol homeostasis, detoxification and processing of xenobiotics, endocrine regulation, blood volume regulation, and immune surveillance). Portal vein supplies around 80% of the blood from the gut to the liver, draining into the hepatic lobules through the hepatic sinusoids [1]. A highly organized liver zonation creates oxygen and metabolic gradients or zones with different and specialized hepatocytes functions. This particular microarchitecture is configured by liver sinusoids, discontinuous and specialized capillaries lined by a fenestrated monolayer of liver sinusoidal endothelial cells (LSECs). The basal side of LSECs interacts with hepatocytes and hepatic stellate cells (HSCs) in the space of Disse, while their luminal side interacts with liver resident immune cells, including Kupffer cells (KCs), hepatic natural killer (NK) cells, and NKT cells and also tissue-resident lymphocytes (T and B cells) [2,3]. KCs are the largest population of tissue-resident macrophages and play an important role in maintaining immune tolerance due to their phagocytic and antigen presentation activity [4]. Likewise, an immunosuppressive environment, an elevated expression of immune checkpoint molecules and an incomplete activation of CD4+ and CD8+ T cells are crucial for an efficient immune tolerance [5]. Intrahepatic innate immune responses are beneficial during acute hepatitis, as the enhance both inflammation and tissue healing. However, sustained immune activation in chronic liver diseases (CLD) may represent the basis for chronic inflammation. The resulting cirrhotic microenvironment promotes the initiation and progression of hepatocellular carcinoma (HCC), despite the underlying molecular mechanisms of the different etiologies [6].

## 2. Cell Death and Inflammation: A Road to HCC

HCC is the most prevalent primary liver cancer and the third most common cause of cancer death worldwide, with a 5-year survival of 18% [7]. HCC is closely associated with chronic inflammation and fibrosis, representing the common end-stage of CLD from excessive alcohol intake, viral hepatitis, and non-alcoholic fatty liver disease (NAFLD) [8]. In this regard, hepatocellular death is a common trigger and sensitive parameter of liver disease progression, and it is well known that specific cell death responses promote liver disease progression through different mechanisms [9]. It has been stated that the relative contribution of cell death to hepatocarcinogenesis depends on the underlying disease. For instance, HBV infection and NAFLD may induce HCC development in the absence of chronic liver injury and/or fibrosis, suggesting alternative cell-death-independent mechanisms that promote carcinogenesis [10]. Nevertheless, it is important to highlight that 80% of HCC occurs in the context of a fibrotic or cirrhotic liver, with significant levels of hepatocellular cell death and inflammation [11].

In liver homeostasis, a balance between the loss and replacement of hepatocytes occurs in a highly regulated manner. Liver regeneration is closely associated with its capacity for xenobiotic and toxin-detoxifying functions after excessive exposure to insults such as food-derived toxins or infections by viruses, bacteria, and/or parasites [12,13]. Therefore, cell death is a crucial feature in chronic inflammatory diseases [14].

Although different modes of cell death have been described, apoptosis and necrosis have traditionally been the most studied and characterized. Apoptosis has conventionally been associated with highly controlled, host-induced cell death in scenarios of injury, representing a key mechanism to prevent malignant transformation. On the contrary, necrosis is still largely considered an “immunogenic” form of cell death. Nevertheless, a growing number of recent studies have shown that specific and distinct programmed cell-death processes, distinct from apoptosis and necrosis, might be involved in the modulation of compensatory proliferation and immune cell activation in CLD [15,16,17].

### 2.1. Apoptosis

Apoptosis is an ordered and orchestrated cellular process that occurs in physiological and pathological conditions. Morphological hallmarks of apoptosis involve DNA fragmentation, plasma membrane blebbing, and cell shrinkage, which lead to cell fragmentation into organelle-containing apoptotic bodies, triggered by activated aspartate-specific proteases, known as caspases [9,18]. Apoptosis is classically considered non-inflammatory or low inflammatory, with a minimal release of damage-associated molecular patterns (DAMPs) due to the rapid removal of apoptotic bodies [19].

**Figure 1:**
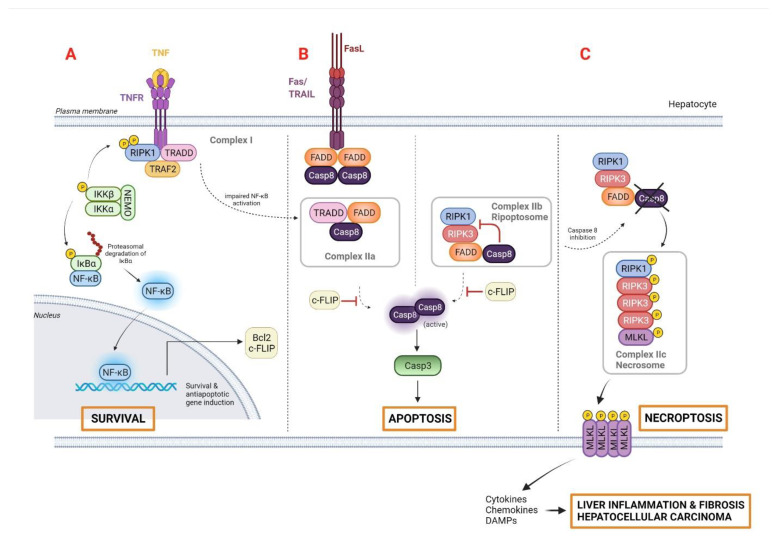
Programmed cell death pathways initiated by TNF and FAS: (**A**) The TNF–TNFR1 cascade simultaneously activates proapoptotic and antiapoptotic signals, and diverse signaling complexes are formed. Complex I, composed by the adaptor protein TRADD, the receptor RIPK1, and the E3 ligase TRAF2, enables the recruitment of the inhibitor NF-ƙB kinase complex, comprising IKKα/β and NEMO. Subsequently, the inhibitor of NF-ƙB (IƙBα) is ubiquitinated and NF-ƙB translocates to the nucleus, promoting the expression of survival and antiapoptotic genes such as Bcl2 and c-FLIP. (**B**) In the FAS/TRAIL-mediated apoptotic pathway, binding of FASL/TRAIL to their cognate receptors results in FAS clustering and binding to FADD. TRADD dissociates from complex I and associates with FADD to form complex IIa, which triggers caspase-8 activation and, consequently, apoptosis. Complex IIb, or ripoptosome, is highly dependent on RIPK1 activity, and represents an alternative death complex that induces apoptosis through direct activation of caspase-8. (**C**) In contrast, caspase-8 can be inhibited under certain physiological or pharmacological conditions, promoting the shift from the ripoptosome-induced apoptosis to necroptosis, a form of programmed necrosis via complex IIc or necrosome. RIPK3 mediates the phosphorylation of MLKL, resulting in its conformational change and oligomerization. This allows MLKL to bind to plasma membrane lipids and form a pore that leads to cell lysis. Release of cellular components (cytokines, chemokines and DAMPs) perpetuates liver inflammation and fibrogenesis in a pro-tumoral microenvironment, thus promoting HCC development. Abbreviations: Bcl2, B-cell lymphoma 2; Casp-3, caspase-3; c-FLIP, cellular FLICE-like inhibitory protein; DAMPs, damage-associated molecular patterns; FAS, FS-7-associated surface antigen; FADD, FAS-associated death domain protein; FASR, FAS receptor; FASL, FAS ligand; MLKL, mixed lineage kinase domain-like protein; NF-ƙB, nuclear factor kappa-light-chain-enhancer of activated B cells; RIPK1, serine/threonine-protein kinase 1; TNF, tumor necrosis factor; TRADD, TNFRSF1A-associated via death domain; TRAF2, receptor-associated factor; TRAIL, Targeting TNF-related apoptosis-inducing ligand. Created with BioRender.com.

Receptor-mediated apoptotic pathways are typically promoted by key regulatory molecules such as the tumor necrosis factor (TNF) family of death receptor ligands (e.g., Fas ligand [FasL]) and TNF-related, apoptosis-inducing ligand (TRAIL), all of which are highly expressed in hepatocytes [20]. Importantly, both mechanisms might be linked, due to the cytochrome c release-mediated activation of caspase-3, the ultimate executioner of apoptosis. The binding of TNF to TNF receptor 1 (TNFR1) induces the formation of complex I, which is involved in nuclear factor-ƙB (NF-ƙB)-dependent expression of pro-survival genes such as the apoptosis regulator B cell lymphoma 2 (Bcl 2) family or the encoding cellular FLICE-like inhibitory protein (c-FLIP), among others (Figure 1A). In fact, TNF-mediated cell death only occurs if NF-ƙB-dependent antiapoptotic signals are suppressed. In that scenario, the lack of inhibition by c-FLIP results in a switch from survival responses towards cell death by the formation of complex IIa or complex IIb, both involved in caspase-8 activation and apoptosis [13]. Complex IIa is formed upon dissociation of the adaptor protein TNFRSF1A-associated via death domain (TRADD) from complex I and its association with FAS-associated death domain protein (FADD), whereas complex IIb or ripoptosome is composed by the receptor-interacting protein kinase 1 (RIPK1), FADD, and caspase-8 (Figure 1B).

Mechanistically, diverse molecules arise as key determinants in apoptotic cell death such as caspase-8 and receptor interacting protein kinase 1 (RIPK1). Recent studies have demonstrated the dual role of caspase-8 in HCC development, triggering apoptosis and promoting cell proliferation by sensing DNA damage in a nonapoptotic function. HCC patients with lower levels of caspase-8 exhibited better overall survival compared with those with high caspase-8 expression. In addition, a significant correlation between caspase-8 and high levels of Ki67 expression was also observed in HCC patients [21]. Importantly, caspase-8 can serve two distinct roles in response to TRAIL receptor engagement. As well as promoting apoptosis through its protease activity, caspase-8 acts as a scaffold for the assembly of a caspase-8-FADD-RIPK1 “FADDosome” complex, leading to NF-ƙB-dependent inflammation, which can be uncoupled from apoptosis and does not require caspase proteolytic activity [22]. This TRAIL-dependent cytokine and chemokine production was observed in different carcinogenic cell types, and an accumulation of tumor-supportive immune cells in the cancer microenvironment has been described in an endogenous TRAIL/TRAIL-R-mediated chemokine (C-C motif) ligand 2 (CCL2) secretion [23].

Regarding the role of RIPK1 in liver cancer, several studies have recently emerged with contradictory results that need to be clarified. On one hand, specific RIPK1 gene deletion in hepatocytes induced the TNF-dependent proteasomal degradation of TNF receptor-associated factor 2 (TRAF2). This mechanism resulted not only in a caspase-8 hyperactivation, but also in an impaired NF-kB activation, promoting the spontaneous development of HCC [24]. On the other hand, another study demonstrated that NEMO, an important signaling adaptor protein in the NF-kB activation pathway, was shown to prevent hepatocarcinogenesis and steatohepatitis by inhibiting RIPK1 kinase activity-dependent apoptosis of hepatocytes [25]. Interestingly, new roles of RIPK1, promoting inflammation, liver fibrosis, and HCC, as well as cell death, have recently been described. In particular, RIPK1 activation is able to promote the transcription of key proinflammatory chemokines such as CCL2, favoring the C-C chemokine receptor type 2 (CCR2)+ macrophage infiltration as well as regulating HSC activation [26]. Similarly, the pharmacological inhibition of RIPK1 reduces necroinflammatory and fibrotic NASH features in HFD-fed mice [27].

Further studies are needed to elucidate the RIPK1 molecular mechanism regulating liver injury. The use of promising RIPK inhibitors, such as necrostatin [28,29], is discussed in the therapeutic approach section.

### 2.2. Necrosis

Traditionally, necrosis has been considered as a dysregulated, accidental, and inflammatory cell death type that culminates in ATP depletion due to mitochondrial dysfunction, loss of membrane integrity, and cell swelling [12,30]. Mitochondrial dysfunction during necrosis occurs due to a prolonged opening of the mitochondrial permeability transition (MPT) pore, which leads to a loss of oxidative phosphorylation and ATP depletion. Necrotic cell death is a significant feature that has been modulated by using JNK and MPT inhibitors in diverse liver injury scenarios [31]. In a mouse model of acetaminophen-induced liver injury, the JNK inhibitor SP600125 completely prevented necrosis when administered after acetaminophen dosing [32]. Similarly, the MPT inhibitor cyclosporine A demonstrated a protective effect in preventing the permeabilization of the mitochondrial membrane and, consequently, reduced hepatic necrotic areas [33].

In the necrosis setting, the release of cellular constituents and DAMPs, such as interleukin (IL)-33 or high-mobility group protein B1 (HMGB1), into the extracellular environment, a process called “oncosis”, elicits a significant proinflammatory response that can also trigger damage to neighboring cells [30]. The non-histone chromosomal protein HMGB1 is considered the prototypical DAMP, released passively by injured or necrotic cells. HMGB1 acts as a proinflammatory cytokine by stimulating necrosis-induced inflammation via toll-like receptor (TLR) 4 activation and the release of cytokines such as IL-8 [34]. Previous studies associated HMGB1 with the prognosis of HCC, suggesting that an overexpression of DAMPs could be a novel, effective, and supplementary biomarker for HCC [35]. Recent data from mouse models have shown that HMGB1 activates ductular reactions via cell-extrinsic mechanisms and RAGE receptor, and promotes hepatocyte transformation [36]. In addition, HMGB1 signaling induces HCC development indirectly through IL-6/Stat3-miR-21-mediated metalloproteinases (MMP) activity [37]. Nevertheless, exactly how DAMPs induce hepatocarcinogenesis remains uncertain, and more studies are needed to unravel this question.

### 2.3. Necroptosis

Necroptosis is the best-studied form of regulated or programmed necrosis, sharing some features with necrosis and apoptosis. Unlike apoptosis, cells undergoing necroptosis show disruptions to their cell membrane, which is a key characteristic of necrosis [38]. Similar to apoptosis, necroptosis uses the same upstream TNF molecular mechanism, resulting in a backup pathway to ensure cell death in situations of apoptosis inhibition [39]. It is well known that activated caspase-8 acts as a crucial executioner of apoptosis, but its suppression might shift the balance to necroptosis [40]. In this scenario, RIPK1, RIPK3, and the mixed lineage kinase domain-like protein MLKL make up the complex IIc, or necrosome, which perforates membrane structures and induces subsequent cell lysis. As a result, necroptosis triggers organelle and cellular swelling and the release of a variety of DAMPs into the extracellular space, eliciting a robust immune response by recruiting different immune cells to heal the damaged tissue [41,42] (Figure 1C).

Several studies have demonstrated the role of RIPK3, activating innate immunity via pattern recognition receptors (PRR) such as NLRPL3 inflammasome activation [43,44]. Apart from the contribution of necroptosis to the inflammatory pro-carcinogenic environment, a direct role of this cell death pathway in HCC has also been studied. Remarkably, the repression of necroptosis was observed in all common human hepatoma cell lines, such as Huh-7, HepG2, and Hep3B, due to a methylation-dependent loss of RIPK3 expression, suggesting that evading this cell death mechanism could be important for the malignant transformation of tumor cells. Therefore, the restoration of RIPK3 expression sensitized cells to chemotherapy, indicating that epigenetic modifications might be an interesting approach to increase chemosensitivity in certain types of HCC [45].

### 2.4. Pyroptosis and the Inflammasome

#### 2.4.1. Pyroptosis

Pyroptosis is a highly proinflammatory cell death mode, predominantly dependent on caspase-1 and caspase 4/5/11 [15,46]. Distinct microbes and host factors activate the pyroptotic cascade, inducing the release of intracellular substances comprising IL-1β, IL-18, IL-33, HMGB-1, and heat shock protein (HSP) through caspase-dependent pore formation, swelling, and rupture of the cell.

**Figure 2:**
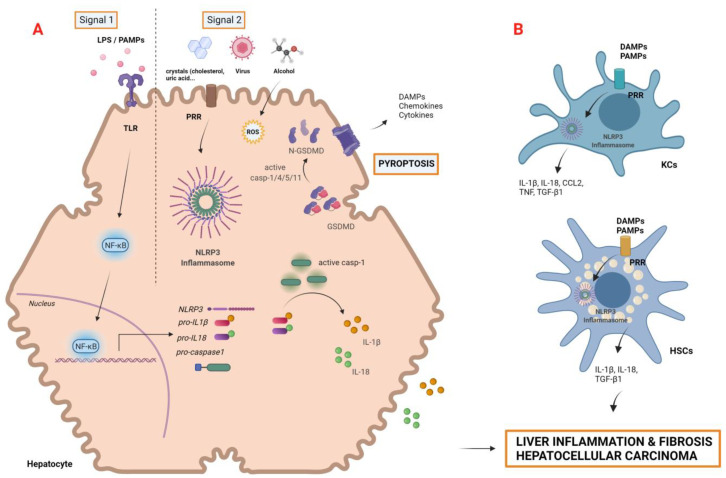
Activation of the NLRP3 inflammasome and canonical pyroptosis in liver cells. Various stimuli of liver damage might cause hepatocyte cell death and gut dysbiosis, leading to high exposure to DAMPs and PAMPs that activate liver inflammasomes: (**A**) In hepatocytes, NLRP3 inflammasome activation requires two steps: the priming signal (SIGNAL 1) is initiated by PAMPs such as LPS, which bind to their corresponding TLR and upregulate the expression of pro-IL-1β, pro-IL-18, pro-caspase-1, and NLRP3 genes via NF-ƙB signaling activation. The second signal (SIGNAL 2) is triggered by PAMPs and/or DAMPs and activates NLRP3, which, in turn, activates pro-caspase-1. Activated casp-1 cleaves IL-1β and IL-18 precursors into mature and proinflammatory forms that are secreted to extracellular space. Casp-1 and, alternatively, caspase-4/5/11 might also cleave protein GSDMD. The N-terminal fragment of GSDMD (N-GSDMD) forms pores in the plasma membrane, inducing pyroptosis by cell swelling and osmotic lysis. (**B**) Inflammasome activation and pyroptosis also occurs in nonparenchymal cells. DAMPSs and gut-derived PAMPs activate Kupffer cells via PRRs, triggering the production of IL-1β and, subsequently, CCL2 and TNF. NLRP3 activation in HSC also induces the expression of the profibrogenic molecule TGF-β. Together, these events result in liver inflammation and fibrosis, perpetuating liver damage and hepatocellular carcinoma. Abbreviations: CCL2, C-C motif chemokine ligand 2; DAMPS, damage-associated molecular patterns; GSDMD, gasdermin D; HSCs, hepatic stellate cells; KCs, Kupffer cells; LPS, liposaccharide; NF-ƙc, nuclear factor kappa-light-chain-enhancer of activated B cells NLRP3, NLR family pyrin domain containing 3; PAMPs, pathogen-associated molecular patterns; PRRs, pattern recognition receptors; TLR, toll-like receptor; TNF, tumor-nuclear factor; TGF-β, transforming growth factor beta. Created with BioRender.com.

Pyroptosis might be triggered by canonical and noncanonical signaling pathways depending on the activated caspase involved. In the canonical pathway, DAMPs and pathogen-associated molecular patterns (PAMPs) are detected by intracellular sensors known as inflammasomes. Inflammasomes are danger-sensing, multimeric protein complexes composed by three elements: (i) A sensor molecule belonging to the NOD-like receptor family such as NLRP3, NLRP1, NLRP4, NLRP6, and NLRP9; (ii) The PYHIN family member AIM2, an adaptor protein with CARD or PYD domains with oligomerization function; (iii) The effector molecule pro-caspase-1 [47]. The most extensively studied and well-characterized inflammasome in liver disease is the NLRP3 complex, which, in response to low-threshold signals, is able to fine-tune the inflammatory response. As shown in Figure 2, the activation of NLRP3 has recently been described as a two-step process. A priming signal (signal 1), resulting from the binding of PAMPs to its cognate TLR, is required to upregulate the transcription of NLRP3, caspase-1, pro-IL-1β and pro-IL-18 genes. Subsequently, a second activation signal (signal 2), provided by DAMPs and/or PAMPs, triggers inflammasome assembly and oligomerization in the cytosol. There, these complexes activate the serine protease caspase-1, which, in turn, induces the maturation of IL-1β and IL-18 and the cleavage of gasdermin D (GSDMD), a pore forming protein that has been recently identified as the ultimate executor of pyroptosis by creating pores in the cell membrane [47] (Figure 2A).

In noncanonical pyroptosis, lipopolysaccharide (LPS), derived from gram-negative bacteria, binds the caspase recruitment domain (CARD) of pro-caspases-4, 5, and 11, promoting the oligomerization and subsequent cleavage of GSDMD. Importantly, in that pathway, the pore in the plasma membrane is formed, but not the cleavage and maturation of proinflammatory cytokines IL-1β and IL-18. However, recent studies have shown that caspase-11 may activate the NLPR3-dependent caspase-1 inflammasome as well, and hence indirectly stimulate the release of intracellular cytokines [48,49].

#### 2.4.2. Inflammasomes in Liver Diseases and HCC

The importance of inflammasomes in liver disease has increased in the last decade, due to its contribution to hepatocyte damage, immune cell activation, and the amplification of liver inflammation [17,50]. Furthermore, several inflammasome receptors (NLRP1, NLRP3, AIM2) are expressed in KCs, LSECs, periportal myofibroblasts and HSCs, participating directly or indirectly in the promotion of liver fibrosis (Figure 2B). One study reported an upregulation of the profibrogenic cytokine transforming growth factor (TGF-β1) in HSCs through the stimulation of inflammasome by uric acid crystals [51]. Similarly, KCs’ inflammasomes can be activated in response to gut-derived PAMPs and hepatocyte-derived DAMPs, producing IL-1β and IL-18, which, in turn, contribute to HSC activation [52].

Increased hepatic levels of caspase-1 and NLRP3 were found in alcoholic liver disease (ALD) patients, and high levels of IL-1β were detected in the serum of severe forms of alcoholic hepatitis [53,54]. Additionally, several studies have shown NLRP3 inflammasome activation in hepatocytes and KCs in the progression of NAFLD [55,56]. Accordingly, the gene expression of pro-IL-1β correlated with the profibrogenic collagen-1 gene in patients with liver steatosis, suggesting an association between NLRP3 inflammasome and the progression from NAFLD to NASH [57].

Although several studies have reported the importance of the NLRP3 inflammasome as a regulator for tumor control, its role in human cancers remains controversial. On one hand, NLRP3 inflammasome activation operates as a pro-tumorigenic factor, promoting proliferation, survival, metastasis, angiogenesis, and immunosuppression [50,58]. For instance, in breast cancer, IL-1β production promotes the infiltration of myeloid-derived suppressor cells (MDSCs) and tumor-associated macrophages (TAMs), providing an inflammatory microenvironment and favoring tumor progression [59]. Although pro-carcinogenic effects have also been described in gastric and prostate cancers, the direct role of NLRP3 inflammasome in liver cancer remains elusive and poorly described. On the other hand, several studies have reported evidence of the protective anti-tumorigenic effects of NLRP3 inflammasome by directly activating pyroptotic cell death or secreting death-inducing cytokines [60]. In colorectal liver metastases, the activation of NLRP3 inflammasome in KCs triggers IL-18 production, stimulating the maturation of NK cells and priming the FasL-mediated apoptosis of tumor cells [61,62]. Concordantly, the expression of all NLRP3 inflammasome components was either completely lost or significantly downregulated in human HCC, showing a significant correlation with advanced stages and poor pathological differentiation [63]. In addition, the reconstitution of NLRP3 inflammasome through the E2/ERβ/MAPK estrogen pathway seems to reverse the malignant phenotype of HCC, reinforcing its tumor-suppressive effect [64].

Given the complex nature of the NLRP3 inflammasome and its seemingly contradictory functions in carcinogenesis, future research needs to address important aspects, such as the driving factors in tumor activation and cross-talk pathways. Targeting the NLRP3 inflammasome, alone or in combination with chemotherapy, may be a promising potential therapeutic approach in cancers.

### 2.5. Autophagy

Autophagy is an intracellular lysosomal pathway that plays a pivotal role in homeostasis maintenance in diverse physiological processes. Considered as a “self-eating” process, capable of resisting metabolic stress by recycling cellular components, autophagy has a controversial dual function in the pathophysiology of liver cancer [65]. The up- and downregulation of this catabolic mechanism was described in HCC, suggesting that autophagy may act as both a tumor promotor and suppressor during malignancy [66]. Furthermore, studies based on the inhibition of autophagy demonstrated that basal autophagy plays a suppressive role in the initial dysplastic stage by maintaining genomic stability, removing damaged mitochondria (mitophagy) and preventing malignant transformation with accumulated mutations [67]. However, once the tumor is established, in a proliferative stage, autophagy would promote tumorigenesis, supporting cell growth [68]. p62, a ubiquitin-binding autophagy receptor, is accumulated in premalignant liver diseases and most HCCs. Its expression is needed and sufficient for activation of the transcription factor NRF2 and mTORC1, the induction of c-Myc, and the protection of HCC-initiating cells from oxidative-stress-induced death [69]. Importantly, autophagy sustains an oxidative metabolism, which is required for tumor development. In a liver damage scenario, active autophagy promotes reactive oxygen species (ROS) generation, resulting in an upregulated oxidative stress that may induce cell death, followed by compensatory cell proliferation [70]. Interestingly, the impact of autophagy-mediated metabolic reprogramming on therapeutic resistance is largely unclear, especially in liver cancer [71,72,73,74]. Sorafenib, an oral multi-kinase inhibitor that is widely used in advanced-stage HCC, results in a significant prolonged survival rate in liver cancer patients [71]. Nevertheless, a considerable number of HCC patients are refractory to sorafenib treatment due to, at least, the potential induction of autophagy through different mechanisms, such as the activation of the AMPK/mTOR signaling pathway or FOXO-3 mRNA methylation and stabilization [72,73,74]. In that sense, strategies focused on autophagy modulation, combined with sorafenib, have been recently described as new therapeutic options with which to overcome drug resistance in HCC [75,76,77].

## 3. Tumor Microenvironment in HCC: The Importance of the Niche

Tumor development and progression regularly occur in concert with alterations in the surrounding stroma, which configures a heterogeneous landscape named the tumor microenvironment (TME). The TME has emerged as an essential driver contributing to tumor survival, growth, angiogenesis or cell invasion, and the maintenance of hepatocarcinogenesis [78]. The TME is orchestrated by cellular and non-cellular components including cancer cells, stromal tissue, and the surrounding matrix. Stromal components comprise immune cells, fibroblasts, angiogenic cells and vascular tissue that, together, actively contribute to elicit inflammatory responses [79]. In chronic liver damage, necroinflammation is characterized by nonparenchymal cell activation and dysregulation, continuous cell death, and regeneration processes leading to fibrosis and tumorigenesis [80]. The crosstalk between hepatocytes and nonparenchymal cells is an emerging paradigm in the pathogenesis of liver diseases [81]. Active communication between stromal cells (recruited or resident), cancer cells and proximal immune cells represent the major point of interest regarding the role of TME in the pathogenesis of HCC by either a direct or indirectly interaction or by the production of inhibitory/stimulatory signals. As heterogeneity of tumor ecosystem cells could also impact the patient’s therapeutic response, TME reprogramming has been intensely explored in recent years. Higher transcriptomic and phenotypic diversity has been described in TME reprogramming, and was associated with poorer overall patient survival [79,82].

Recent comprehensive studies have made efforts to describe the immune landscape of HCC. Single-cell approaches have identified diverse immune-subtypes from the adaptive and innate immune system, with important functions in immunosuppression, immunocompetence, and immunodeficiencies in hepatic tumors [4]. This review is particularly focused on the contribution of innate immune cells and hepatic nonparenchymal cells to HCC development.

### 3.1. Myofibroblast-Derived Cells and the Matrisome

**Figure 3:**
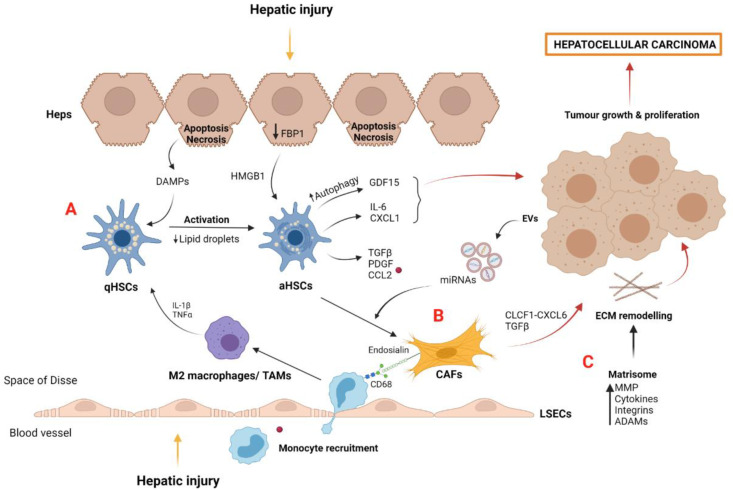
Myofibroblast-derived cells and matrisome contribution to tumor microenvironment and HCC development: (**A**) Quiescent HSCs become activated by DAMPs released by apoptotic hepatocytes and by IL-1β and TNFα secreted by polarized M2 macrophages after hepatic injury. aHSCs induce the secretion of proinflammatory growth factors and chemokines such as CCL2, which is recognized by macrophage CCR2, perpetuating liver damage and tumor growth. aHSCs, induced by the loss of hepatocyte FBP1, secrete cytokines such as IL-6 and CXCL1, which are involved in liver tumorigenesis. Growth factor GDF15, induced by aHSC autophagy, also contributes to hepatocarcinoma. (**B**) CAFs are derived from activated myofibroblasts such as aHSCs. Diverse growth factors and miRNAs contained in EVs and released from cancer cells act as positive stimuli for CAF differentiation. CAFs promote tumor development via the CLCF1–CXCL6/TGFβ signaling pathway and induce the recruitment of macrophages and M2 polarization by endosialin–CD68 interaction. (**C**) The matrisome plays a key role in hepatocellular carcinoma development by the action of several molecules secreted into the tumor microenvironment, such as MMP, cytokines, integrins, and ADAMs. Abbreviations: aHSCs, activated hepatic stellate cells; qHSCs, quiescent hepatic stellate cells; CAFs, cancer-associated fibroblast cells; CCL2, chemokine (C-C motif) ligand 2; CLCF1, cardiotrophin like cytokine factor 1; ECM, extracellular matrix; EVs, extracellular vesicles; Heps, hepatocytes; HGF, hepatocyte growth factor; HMGB1, high mobility group box protein 1; LSECs, liver sinusoidal endothelial cells; MMP, metalloproteinases; TAMs, tumor-associated macrophages; PDGF, platelet-derived growth factor; TAMs, tumor-associated macrophages; TGF-β, transforming growth factor beta. Created with BioRender.com.

HSCs are a sinusoidal cell type that is mainly involved in ECM production and deposition, triggering fibrosis development [83]. Upon liver injury, HSCs become activated by DAMPs secreted by apoptotic hepatocytes and paracrine signals from LSECs and immune cells [81,84] (Figure 3A). Autophagy also plays a key role in HSC activation, partly by lipid droplet digestion favoring progression of liver diseases [85,86]. In this sense, a recent study described the implication of autophagy in HSCs promoting HCC progression [87]. Specifically, in a cell culture system resembling a tumor microenvironment, activated HSCs (aHSCs) showed upregulated levels of growth differentiation factor 15 (GDF15) compared to the inhibited autophagy condition. Accordingly, single-cell RNA sequencing of tumor and non-tumor tissues from HCC patients revealed that HSCs from tumoral areas showed a significant increase in GDF15 expression, suggesting the key role of this protein in tumor progression.

aHSCs also produced several proinflammatory cytokines and growth factors, such as TGFβ and PDGF, among others [88]. Analysis of peritumoral and tumoral tissues from an HCC cohort showed a specific gene profile in peritumoral aHSCs that predicted tumor recurrence and patients’ survival, highlighting their contribution to the tumor microenvironment. Mechanistically, aHSC showed an upregulation of CCL2 when co-cultured with CCR2+ monocytes, a myeloid cell responsible for reprogramming towards an M2 immunosuppressive and tumor-promoting phenotype [89].

During liver disease progression, aHSC becomes senescence and may switch from a profibrogenic to proinflammatory phenotype [90]. In a murine NAFLD-HCC model, loss of hepatocyte FBP1, a gluconeogenic enzyme, induces HSC activation via HMGB1, followed by the release of senescence-associated secretome components such as IL-6 and CXCL1, promoting liver tumor growth [91]. Importantly, these results reinforced the idea of crosstalk between HSCs and hepatocytes in liver tumorigenesis [92]. Additionally, the important bidirectional communication between HSCs and macrophages in liver damage progression has been demonstrated. Macrophages release profibrogenic mediators such as TNFα and IL-1, which activate HSCs [93], whereas aHSCs secrete chemokines such as CCL2, which is recognized by the CCR2 of macrophages and monocytes, promoting their recruitment and aggravating liver injury [94] (Figure 3A).

Tumors also contain other myofibroblastic stromal components, including cancer-associated fibroblasts (CAFs). It is considered that CAFs derive from pre-existing activated myofibroblasts through interaction with cancer cells (Figure 3B). CAFs contribute to tumor development and progression via crosstalk through soluble factors and extracellular vesicles (EVs) [95,96]. In this regard, microRNAs contained in EVs derived from liver cancer cells have been recently described as responsible for the formation of the microenvironment in metastases [97]. Moreover, CAFs may communicate with liver cancer cells via the cardiotrophin-like cytokine factor 1 (CLCF1)–CXCL6/TGFβ axis, consequently stimulating the development of the stem-cell-like tumor phenotype and the recruitment and polarization of neutrophils, altogether aggravating HCC prognosis [98].

CAFs are characterized by the secretion of high amounts of collagen in the HCC stroma or ECM, and the upregulation of the chemokine CXCL12, also known as stromal-cell-derived factor 1 (SDF-1), which stimulates tumor growth and neo-angiogenesis by the recruitment of endothelial progenitor cells [99]. A recent comprehensive study has shown that CAFs recruit macrophages through endosialin–CD68 interaction, stimulating the pro-oncogenic or M2 polarization of macrophages [100]. Furthermore, the authors found that endosialin expression was inversely correlated with patient prognosis, and results from in vitro and in vivo experiments suggested the endosialin–IgG78–GAS6 axis as an important mechanism in HCC progression.

In the tumor context, the stroma is characterized by ECM remodeling and stiffness, which are necessary processes for tumor growth and angiogenesis. Matrisome represents all the components of ECM, including collagen and transmembrane proteins, ECM regulators such as MMPs, and several cytokines that are secreted and deposited in ECM. The liver matrisome provides support and structure to cells, as well as providing an important reservoir of molecular factors involved in cell communication [101]. The effect of MMPs is essential in ECM degradation, facilitating tissue invasion by cancer cells and new capillary vessel formation, known as sprouting angiogenesis [102]. Likewise, the ECM of the fibrotic premalignant environment perpetuates advanced liver disease and HCC tumor migration through the upregulation and secretion of MMPs [103] (Figure 3C).

Integrins are the main adhesion molecules and mechano-sensors that allow for communication between ECM and cell signaling pathways, stimulating cytoskeletal remodeling [104]. They are composed of α and β subunits, which interact with ECM proteins such as vinculin and tallin, and cooperate with specific growth factor receptors. The interaction between integrins and the TGFβ signaling pathway regulates the cellular response at different stages of HCC. Accordingly, at the early stages of liver cancer, integrins inhibit TGFβ-induced apoptosis through MEK/ERK activation [105], whereas, at later stages, they cooperate with TGFβ to regulate tumor proliferation and invasion [106]. Moreover, β1-integrin was associated with the regulation of stiffness in human HCC cells, inducing tumor proliferation [107].

On the other hand, adamalysins (ADAMs), disintegrins, and MMP, which are involved in ECM remodeling, have been also associated with HCC development (Figure 3C). In this sense, ADAM10 was upregulated in human HCC tissue compared with the adjacent non-tumor tissue, and positively correlated with poor prognosis and the shorter survival of HCC patients [108]. Moreover, in vitro experiments demonstrated ADAM10 implication in cell proliferation and invasion. Another study demonstrated that ADAM12 RNA was increased in human activated HSCs associated with ECM accumulation during liver injury [109]. In fact, an upregulation of ADAM12 was detected in cirrhotic and HCC liver tissue and correlated with MMP2 expression and activity, highlighting the significance of ADAM12 in matrix remodeling. Several studies have suggested the important contribution of ADAM proteins in hepatocarcinogenesis, suggesting their use as potential therapeutic targets for HCC. For instance, inflammatory cytokines such as TNFα and IL-6 are directly regulated by ADAM17, with this interaction perpetuating liver inflammation and HCC progression [110].

### 3.2. Macrophages and Tumor-Associated Macrophages (TAMs)

**Figure 4:**
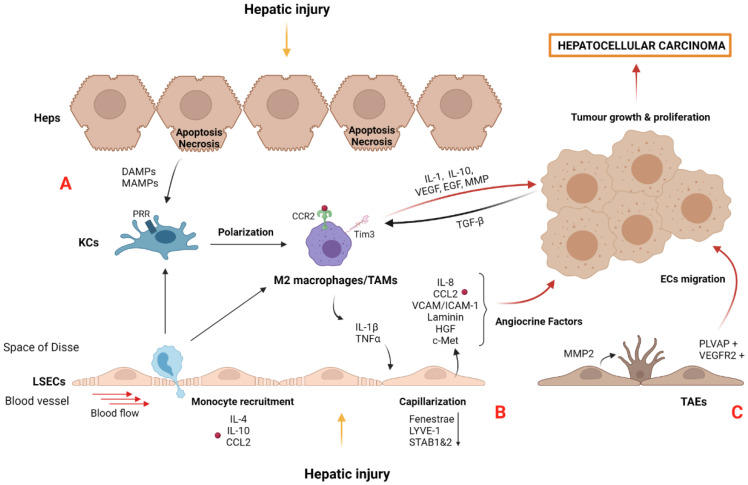
Liver damage activates and dysregulates liver macrophages and LSECs’ role in HCC: (**A**) KCs are polarized to TAMs, a M2 phenotype macrophage with proangiogenic and proinflammatory properties that act on neighboring cells. Moreover, DAMPs and MAMPs are recognized by PRR on KCs aggravating liver diseases and hepatocellular carcinoma development. Importantly, exhausted KCs are replaced by recruited circulating monocytes, which polarize to TAMs. TAMs can also be recruited through IL-4, IL-10, and CCL2 signals from circulating monocytes. Crosstalk between immune and cancer cells occurs through TGFβ-Tim3 interaction and promotes the polarization of M2 TAMs towards a pro-oncogenic phenotype that triggers tumor growth, invasion, and migration by several secreted factors such as cytokines, proangiogenic factors, and MMPs. (**B**) Liver damage causes LSECs capillarization, losing specific features such as fenestration and homeostatic proteins (LYVE-1 and STAB1 and 2). Subsequently, LSECs secrete several proinflammatory (IL-8 and CCL2) and adhesion molecules (VCAM-1 and ICAM-1), ECM components (laminin) and growth factors (HGF) that act as angiocrine factors favoring the capillarization process and tumor microenvironment during tumorigenesis. (**C**) PLVAP+ and VEGFR2+-ECs migrate to tumoral tissue contributing to tumor growth by the formation of new vessels. Moreover, TAEs promote neo-angiogenesis by upregulation of MMP2, inducing ECM remodeling. Abbreviations: CCL2, chemokine (C-C motif) ligand 2; CCR2, C-C chemokine receptor type 2; DAMPS, damage-associated molecular patterns; Heps, hepatocytes; ECs, endothelial cells; ICAM-1, intracellular adhesion molecule 1; KCs, Kupffer cells; LSECs, liver sinusoidal endothelial cells; LYVE-1, lymphatic vessel endothelial hyaluronan receptor 1 MAMPs, microbial-associated molecular patterns; PLVAP, plasmalemma vesicle associated protein; PRRs, pattern recognition receptors; STAB, stabilin; TAEs, tumor-associated endothelial cells; TAMs, tumor-associated macrophages cells; VCAM-1, vascular cell adhesion molecule 2. VEGFR2, vascular endothelial growth factor receptor 2. Created with BioRender.com.

Macrophages within the liver consist of KCs, the resident liver macrophages, and monocyte-derived macrophages recruited in an inflammatory situation [111]. DAMPs released from apoptotic hepatocytes, such as hepatocyte DNA and microbial-associated molecular patterns from gut dysbiosis are, recognized by the PRR of KCs, resulting in phenotype alteration and liver disease aggravation [112] (Figure 4A). Dysbiosis contributes to increased gut permeability, altering the bacterial transit to the liver. In this regard, LPS, a gram-negative bacteria component, is recognized by TLR4 expressed in the surface of KCs, resulting in proinflammatory cytokine secretion such as IL-1 and TNFα via the MyD88 signaling pathway [113,114]. For these reasons, knowledge of the microbiome and its metabolites have recently been proposed as a new prognosis tool during HCC development [115].

Interestingly, KCs become exhausted in an HCC microenvironment. Nonetheless, recruited monocytes contribute to their replacement by acquiring a KC-like phenotype [116]. Crosstalk between sinusoidal liver cells is not only essential in this renewal process, but also represents an important mechanism for KC replacement in CLD and HCC. Indeed, Bonnardel et al. reported that KC death promoted the recruitment of circulating monocytes and activated HSCs and LSECs via the LXR-α-NOTCH-BMP signaling pathway [93].

The transition of proinflammatory M1 macrophages to M2 TAMs with anti-inflammatory but pro-oncogenic properties is mediated by different cytokines (IL-4, IL-13, and IL-10) and molecular signals (glucocorticoids, vitamin D3) produced by tumor or stromal cells [117]. Recently, macrophage polarization has been related to matrix stiffness and HCC development [118]. Results from an in vitro study using a gel-based culture system with tunable stiffness demonstrated that there was a correlation between high stiffness, characteristic of advanced CLD, and HCC, with remarkable M2 polarization via the upregulation of HIF-1α and LOXL2. In this sense, matrix stiffness in CLD and HCC promotes a stiff environment and perpetuates liver damage [107,119]. Macrophage cell death mechanisms further contribute to tumorigenesis. In this regard, autophagy-silenced KCs isolated from a DEN-induced hepatocarcinogenesis model increased proinflammatory and profibrogenic factors such as IL-1α/β and TGFβ, contributing to HSC dysregulation. This was accompanied by ROS accumulation in mitochondria and the activation of NLRP3 inflammasomes [120].

TAMs are defined as macrophages infiltrating tumor tissues or populating the surrounding TME. They play a key role in the creation of an immunosuppressive TME and exhibit important functions in regulating metastasis. TAM precursors are recruited into the TME as circulating monocytes through IL-4, IL-10, and CCL2 signals, among others [95,117] (Figure 4A). TAMs adopt an immunosuppressive and pro-oncogenic phenotype that expresses a set of genes including APOE, TREM2, and the transcription factors ID3 and MAF [121]. Importantly, SLC40A1 and GPNMB were found to be expressed in hepatic TAMs, and in vitro experiments validated their association with a poor prognosis of HCC.

TAMs suppress anti-tumor immunity by the secretion of chemokines and cytokines such as IL-1 and IL-10, proangiogenic factors such as VEGF and EGF, and MMP including MMP-9 [117]. These secreted molecules have been implicated in several pro-tumorigenic processes including tumor invasion and growth and angiogenesis [122]. In that sense, high amounts of CD163+ TAMs from peritumoral human liver tissue were correlated with poor prognosis and an increased number of tumor nodules in HCC patients. These results were validated in both an orthotopic tumor model and in vitro experiments demonstrating that TAMs contributed to tumor growth and invasiveness [123]. Moreover, tumor growth and poor prognosis were also associated with an increased Tim-3 expression in TAMs from HCC patients. Similarly, TGFβ released by cancer cells induced TAMs polarization via Tim-3, as well as increased cytokine secretion, which favored HCC development [124] (Figure 4A).

A transcriptomic analysis of human tumor liver tissue showed an increased expression of several chemokines, such as CCL20, CXCL10, and CXCL11, compared with adjacent non-tumor liver tissue [125]. The CCL20 receptor, known as CCR6, was highly expressed in circulating macrophage and monocyte populations from HCC patients, and the number of intratumoural TAMs showed a positive correlation with CCL20 mRNA expression levels. In addition, TAMs expressed higher levels of IL-10 in tumor compared with non-tumor liver tissue. Together, these results highlight the important role of chemokines secreted by TAMs, favoring the immunosuppressive tumor microenvironment.

### 3.3. Liver Sinusoidal Endothelial Cells and Tumor-Associated Endothelial Cells (TAEs)

A healthy microenvironment within the sinusoids is disturbed after prolonged injurious stimuli, such as viral infection, high-fat diet, and/or alcohol consumption [83]. LSECs shape the open barrier between liver sinusoids and parenchymal cells. Phenotypic alterations in LSECs occur from the initial stages of liver injury to HCC development through a process known as capillarization (Figure 4B). In this scenario, LSECs lose their characteristic transmembrane pores (fenestrae) and acquire vasoconstrictor, proinflammatory, prothrombotic, and pro-tumorigenic properties [3,126]. At the molecular level, LSECs lose the expression of homeostatic proteins such as LYVE-1 and STAB1 and 2 [127].

The capillarization process might be induced by stimulation with proinflammatory cytokines, including IL-1β and TNFα, secreted by infiltrated immune cells, contributing to chronic inflammation and tumorigenesis [93,128]. Furthermore, capillarized LSECs release proinflammatory molecules such as cytokines (IL-8 and MCP1) and express adhesion molecules (VCAM-1, ICAM-1, and E-selectin) that act as angiocrine factors supporting the immunosuppressive microenvironment and tumor development [129,130,131]. Recently, the role of ICAM-1 in the interaction of tumor cells with LSECs has been described [132]. Primary isolated LSECs co-cultured with colon adenocarcinoma cells increased the secretion of ICAM-1 and other proinflammatory molecules such as IL-1β and TNFα. Consequently, tumor cell adhesion and transmigration were enhanced, promoting tumor metastasis. In that sense, a clinical study analyzing patients with CLD from different etiologies reported the association of soluble ICAM-1 with an advanced tumor stage in HCC [133]. Capillarized LSECs also produced ECM components, such as collagens, fibronectin, and laminin that perpetuate liver damage [134], and were replaced by undifferentiated ECs in a yes-associated protein (YAP)-induced hepatocarcinogenesis model [135]. Specifically, YAP+ tumor cells increased osteopontin secretion, an important driver of crosstalk between endothelial cells through the hepatocyte growth factor (HGF)/c-Met pathway, promoting tumor cells’ proliferation and survival [136] (Figure 4B).

Once the tumor is established, ECs not only form the blood vessels that supply oxygen and nutrients to tumoral cells, but also communicate with them via angiocrine factors such as HGF and ICAM-1, which promote tumor progression [137]. It has been demonstrated that the Notch1 signaling pathway was activated in the ECs of different tissues contributing to tumor cell migration and colonization in the liver [138]. In contrast, Notch1 was described as an important signaling pathway for vascular homeostasis, maintaining LSECs phenotype, and cell survival [139]. Indeed, Notch1 knock-out mice showed intussusceptive angiogenesis, an alternative form of angiogenesis associated with CLD and hepatocellular carcinoma development. Despite these controversial findings, these studies revealed the key role of Notch1 in the endothelial dysregulation.

Recently, single-cell sequencing data have shown that tumoral and adjacent non-tumoral liver tissues are enriched with TAMs and TAEs. Specifically, a different phenotypic profile has been described in endothelial cells (ECs), depending on their location. Intratumoral TAEs are characterized by the expression of PLPP3, IGFP3, VEGFR2, and PLVAP, whereas adjacent non-tumoral tissue are represented by CD9 and CD320 positive endothelial cells [140].

It is well known that ECs play a key role in neo-angiogenesis by actively contributing to tumor growth through cell migration from adjacent non-tumor to tumor tissue. In this regard, the migration of ECs into the tumor has been described using RNA velocity analysis [140] (Figure 4C). PLVAP+ ECs were also identified in different peripheral clusters, suggesting that they originally formed an intermediate subpopulation that migrated into the tumor core. Moreover, the tumoral ECs’ cluster expresses VEGFR2, a molecule that facilitates the communication of blood vessels with tumor hepatocytes, promoting angiogenesis [141]. A comparative study of TAEs with LSECs demonstrated phenotypic differences related to angiogenesis modulation. TAEs promoted the formation of new vessels through MMP2 upregulation, thereby causing the degradation of the ECM [142,143].

## 4. Targeting Cell Death and Tumor Microenvironment

HCC is the second leading cause of cancer-related death worldwide, with an increased burden that may soon exceed one million cases per year [10]. Treatment of HCC is challenging because it is a complex, multistep process, which is highly resistant to therapy [144]. Therefore, the therapeutic approach may vary depending on the stage of liver cancer at the moment of HCC diagnosis [145]. For instance, sorafenib and lenvatinib are first-line systemic treatments that target both cancer cells and cells from the TME by inhibiting up to 40 kinases, including VEGF receptors, PDGF receptors, and diverse cell proliferation drivers such as KIT and RAF1 [144,146].

Targeting cell death by preventing or reducing hepatocyte apoptosis and/or other forms of cell death could be considered to disrupt the inflammation–fibrosis–HCC axis and prevent CLD progression (Table 1). Pan-caspase inhibitors such as emricasan have been investigated in CLD, with controversial results. While preclinical studies demonstrated beneficial effects in fibrosis and portal hypertension [147,148], recent clinical trials showed no effects for either reducing portal hypertension [149] or improving fibrosis in patients with NASH and F1-F3 fibrosis [150]. The efficacy of different caspase inhibitors in patients with liver damage should be tested, especially if necroptosis events could be induced when apoptosis was blocked [151,152,153]. Thus, the inhibition of RIPK1 kinase activity could be a good therapeutic option, not only by reducing apoptosis but also by modulating necroptosis [25,154,155]. Importantly, phase I clinical trials showed that RIPK1 inhibitors were well-tolerated in humans and mice [156,157]. Alternatively, patients with an established HCC could benefit from the increased sensitivity of liver tumor cells to cell death. In this regard, drugs such as second mitochondria-derived activator of caspase (SMAC) mimetics, which target the inhibitor of apoptosis proteins (IAP), might reduce tumor growth by inducing cell apoptosis [158], and also sensitize HCC cells to cytokines or immune-cell-mediated cell-killing, a strategy known as cytotoxic therapy [159]. On the other hand, COX-2 inhibitors, such as the antifungal ketoconazol, decreased COX-2 expression, resulting in Parkin mitochondrial translocation and excessive mitophagy activation in a preclinical model of HCC, together contributing to HCC cell apoptosis and tumor suppression [160].

Targeting the inflammasome in CLD has been widely studied in recent years. MCC950 is a selective NLPL3 inhibitor that improves liver injury, reduces liver macrophage and neutrophil infiltration, and modulates fibrotic progression in experimental NASH models [161,162]. Moreover, the pharmacological inhibition of P2X7R, one of the most potent activators of the NLRP3 inflammasome, results in a significant protection from inflammation and fibrosis in a liver damage animal model, not only by directly reducing the inflammatory infiltrate and collagen deposition, but also by indirectly impairing IL-1β-mediated-hepatocyte cell death [163].

Some studies have reported a tumor-suppressor function of NLRP3 inflammasome activation through an increase in pyroptotic cell death [164]. 17β-estradiol (E2) and estrogen receptor (ER) signaling could protect against HCC, not only by triggering this caspase-1-dependent mechanism, but also by inhibiting the beneficious effects of autophagy in tumoral cells [165]. These findings might be interesting in a chemotherapy-resistance setting, as autophagy has been described as a chemoresistance mechanism of cancer cells against cytostatic drugs such as sorafenib [166,167]. Alternatively, directly targeting the inflammasome activation in tumoral cells might also represent a potential therapeutic option.

Different inhibitory compounds such as luteoloside and anisodamine have been shown to have antitumoral activity in vitro and in vivo, including the suppression of cell proliferation and the impairment of cell migration and invasion [168,169]. Further investigations are needed to better understand the precise mechanisms underlying inflammasome activation and to design inflammasome-directed therapies for CLD and HCC.

As abovementioned, the majority of HCC occurs in the context of liver cirrhosis. Therefore, the dysregulation of liver cells is a key point for the development of new therapies aimed at slowing/stopping the progression of CLD to advanced cirrhosis. LSECs are one of the most important cell types in the tumor microenvironment, supporting the progression of metastasis. The administration of miR-20a-conjugated nanoparticles in a mouse model of colorectal cancer liver metastasis reduced the metastatic tumor-occupied area and the protein expression of genes involved in migration and differentiation, such as E2F1 and ARHGAP1 [170]. The transcriptional regulator GATA4 has also been recently reported as a master regulator of LSEC differentiation, modulating the profibrotic angiocrine switch, and making it an attractive therapeutic option [171]. Other experimental approaches have been designed to repress tumor-driven angiogenesis. Specific therapeutic strategies against the VEGF–VEGFR2 axis were designed to block the activation of TAEs. Anti-VEGFR2-targeted immunoliposomes loaded with doxorubicin, a chemotherapeutic drug, reduced blood microvessel density promoting endothelial apoptosis and impairing tumor cell proliferation [172]. Bevacizumab, a recombinant humanized monoclonal antibody developed against VEGF was the first antiangiogenic drug introduced into clinical practice that blocks blocking TAE activation in newly formed tumor vessels [173]. However, more recent studies focused not only on inhibiting tumor angiogenesis, but also on restoring the host immune function against the tumor. Thus, the combination of anti-VEGF and anti-PD1/PD-L1 therapies emerge as a promising approach for the treatment of HCC [174,175,176].

Promoting programmed cell death in aHSC has been used to offer an alternative strategy for the amelioration of liver fibrosis. Curcumol, a guaiane-type hemiketal extracted from *Rhizoma Curcumae*, induces RIPK1/RIPK3 complex-dependent necroptosis via JNK1/2-ROS signaling in hepatic stellate cells and decreases hepatic fibrogenesis [177]. Likewise, gallic acid, a natural phenolic acid, induces necroptosis in vitro in aHSC via the TNF-α signaling pathway [178]. Alternatively, the possibility of targeting different ECM components to improve drug response has also been explored. Beta-aminopropionitrile fumarate (βAPN), an inhibitor of all five lysyl oxidase family members, was used to treat established tumors in different syngeneic mouse models by interfering with collagen stabilization. The results showed an improvement in tumor supply and an inhibition of tumor growth [179]. Similarly, small molecules have also been developed to treat hyaluronan-rich tumors and to increase uptake and efficacy of chemotherapeutic agents. Treatment with 4-MU, a hyaluronan synthases inhibitor, significantly reduced hyaluronic acid accumulation in tumor-bearing mice, suppressed human pancreatic cells, and suppressed the cellular proliferation, migration, and invasion of human pancreatic cells in in vitro assays [180].

Chemokines are important mediators in chronic inflammation, fibrogenesis and immune cell infiltration [181]. Several studies have reported that blocking chemokine receptors mitigates liver inflammation and fibrosis in preclinical studies [182]. In this regard, the CCL2–CCR2 axis could be a perfect target for CLD progression. In vivo and in vitro experiments have demonstrated the beneficial effects of targeting the CCL2–CCR2 signaling pathway on liver inflammation, including the deactivation of aHSCs and the reduction of macrophage infiltration and angiogenesis [94,183,184,185]. Elevated serum concentrations of cytokines, including chemokines, interleukins, and TNF or TGF-β, secreted during CLD, suggest the important implication of these molecules in HCC development. The blockade of TGF-β significantly decreased tumor growth and metastasis by decreasing the collagen I content and normalizing the tumor stroma in two mammary carcinoma models. The pharmacological inhibition of TGF-β with the monoclonal antibody 1D11, and the genetic overexpression of sTβRII, a soluble TGF-β type II receptor, improved tumor vessel perfusion and intratumoral distribution of chemotherapy drugs [186]. A recent study observed that the combination of sorafenib and galunisertib, a TGF-β receptor type I inhibitor responsible for reducing the growth, invasion, and progression of HCC, showed a prolonged overall survival with acceptable safety in patients with advanced HCC [187]. However, additional studies reporting different therapeutic options targeting cytokines derived from aHSC, capillarized LSEC, or polarized hepatic macrophages, in the context of advanced CLD progression to HCC development, are still needed.

Once the tumor is established, stromal cells represent an interesting therapeutic target. Preclinical studies using murine HCC models have suggested that targeting the the CCL2–CCR2 and CXCL12–CXCR4 axes may be a useful strategy to prevent tumorigenesis [188,189]. The use of BPRCX807, a highly selective CXCR4 antagonist, reduced TAM polarization and recruitment, and decreased angiogenesis, tumor metastasis, and proliferation through AKT- and ERK-signaling inhibition [190]. Recently, WSX1, a receptor subunit for IL-17, has been identified as a tumor-suppressor in hepatocytes, and was proposed as a potential target for immunotherapy [191]. In this context, osteopontin, described as a prominent mediator in the immunosuppressive and inflammatory microenvironment, has been suggested as a new immunotherapy strategy in combination with anti-PD-1/PD-L1-based therapies [192]. The combination of both strategies reduced macrophage infiltration, M2-TAM polarization, and inhibited tumor growth.

**Table 1:** Therapeutic strategies targeting cell death and tumor microenvironment.

Target	Therapeutic Strategy	Pre-Clinical Model	Effects in Liver	References
**Cell Death**				
Apoptosis and necroptosis	Pan caspase inhibition (Emricasan)	CCl_4_-cirrhotic rats: 10 g/kg/day	Reduced portal hypertension and liver fibrosis.	[147,148]
	Caspase-8 loss	Acute liver injury in Casp8^Δhepa^ mice	Protection from hepatocarcinogenesis.	[153]
	RIPK1 activity inhibition	RIPK1 ^D138N/D138N^ mice	Prevented steatohepatitis and HCC by cell death inhibition. Amelioration of liver inflammation and prevented HCC by hepatocyte cell death protection.	[25]
	Caspase-3 activation (Smac mimetic BV6 and oleanolic acid)	In vitro (HCC cell lines, MTT assay): 60 µM OA and/or 4–6 µM BV6 In vivo (Chorioallantoic membrane assay): 30 µM OA and/or 4 µM BV6	Induction of hepatocarcinogenic cell death.Suppressed tumor growth.	[158]
	Caspase-3/8/9 activation (Smac mimetic APG-1387 and TNF)	In vitro (HCC cell lines, colony forming assay): 2 μM APC-1387 and 100 ng/mL TNFα In vivo (subcutaneous xenograft tumor model): 20 mg/kg	Cell death induction and sensitization to natural killer cell-mediated cell killing.	[159]
	Mitophagy-mediated apoptosis (Ketoconazole)	In vitro (HCC cell lines, MTT assay): 20 μM In vivo (xenograft tumor model): 20 μM	In vitro: inhibition of cell proliferation. In vivo: inhibitory effect on tumor growth.	[160]
Inflammasome and pyroptosis	NLRP3 inhibition (small molecule MCC950)	NASH mouse model: 20 mg/kg	Reduced liver inflammation and fibrosis.	[161]
		In vitro (primary hepatocytes): 50 μM	Abrogation of pyroptotic cascade in steatotic hepatocytes.	[162]
	NLRP3 inhibition (pharmacological P2X7R inhibitor SGM-1019)	In vitro (primary human Kupffer Cells): 1 μM In vivo (liver fibrosis non-human primate model): 10 mL/kg	Reduced IL-1β production. Protection against liver inflammation and fibrosis.	[163]
	NLRP3 inhibition (Luteoloside)	In vitro (HCC cell lines): 50 μM In vivo (xenograft tumor and metastasis model): 2 mg/kg body	In vitro: blockade of HCC cell migration and invasion. In vivo: inhibition of proliferation and metastasis in HCC.	[168]
	NLRP3 inhibition (Anisodamine)	In vivo (xenograft tumor model): 10–200 mg/kg	Suppressed HCC cells growth, induced apoptosis, and regulated the levels of inflammatory factors.	[169]
	NLRP3 activation (Alpinumisoflavone)	In vitro (HCC cell lines, proliferation, migration, and invasion assays): 0–20 μM In vivo (xenograft HCC model): 20 or 40 mg/Kg	In vitro: suppressed cell proliferation, migration, and invasion capacity.In vivo: suppressed tumor growth.	[164]
	NLRP3 activation (17β estradiol/E2)	In vitro (HCC cells): 50–100 nM	Induced pyroptotic cell death and inhibition of protective autophagy.	[165]
Autophagy	Autophagy cell death (Ipatasertib GDC0068)	In vitro (HCC cells): 1–10 μM In vivo (subcutaneous xenograft tumor models): 25 mg/kg	Suppressed sorafenib-resistant HCC cells growth by inducing autophagic cell death.	[166]
	Autophagy cell death (SC-59)	In vitro (HCC cells, MTT assay): 10 μM In vivo (subcutaneous xenograft tumor model): 20 mg/Kg	Inhibition of tumor growth.	[167]
**TME** **reprogramming**				
Stromal components and inflammation	Tumor-infiltrated LSECs reduction (Nanoparticle-mediated delivery of miR-20)	In vitro (migration assay) In vivo (liver murine metastasis model) 16.7 lg/mL miR-20	Reduced activated LSEC recruitment into metastatic foci. Decreased liver metastasis progression.	[170]
	Blocking tumor-associated endothelium (TAEs) (Liposome-mediated delivery of anti-VEGFR2 mAb DC101)	In vitro (MS-1 mouse endothelial cells HT-29 human colon cancer and MDA-MB-468 human breast cancer cell) In vivo (Insulinoma model Rip1Tag2 and breast cancer model MMTV-PyMT transgenic mice)protein/liposome ratio of 60 μg Fab’/μmol PL	Reduction in blood vessel density. Inhibition of tumor growth.	[172]
	Induction of aHSC necroptosis (Curcumol)	In vitro (LX2 HSC line) 30 µM In vivo (murine CCl4 fibrosis model)	Inhibition of HSC activation. Reduction of inflammatory cell infiltration and fibrosis amelioration.	[177]
	Induction of aHSC necroptosis (Gallic Acid)	In vitro (rat primary HSC): MTT assay, proliferation assay, DNA oxidative damage detection (50–75 µM)	Induction of oxidative stress, cytotoxicity, and programmed necrosis in aHSC.	[178]
	Interfering collagen stabilization (βAPN: Three-aminopropionitrile fumarate)	In vivo (mice breast adenocarcinomas engraftment): 100 mg/kg BW	Improvement of tumor supply. Inhibition of tumor growth.	[179]
	Inhibition of glycosaminoglycan (HA) synthesis (4-MU)	In vitro (MIA PaCa-2 human pancreatic cells): Proliferation assay, wound healing assay, invasion assay: 0.5 mM MU In vivo (intra-abdominal cell cancer implantation): 2 mg/g BW	Reduced pericellular matrix containing HA. Inhibition of cell proliferation, migration, and invasion of cancer cells. Improved survival rates.	[180]
	Blocking TGF-β (TGF-β–neutralizing antibody, 1D11 or Genetic overexpression sTβRII)	In vivo (orthotopic mouse model of mammary carcinoma):1D11 (5 mg/kg)	Improvement of tumor vessel perfusion. Enhancing an intratumoral distribution of chemotherapy drugs.	[186]
	Blocking circulating monocyte recruitment (CCL2 inhibitor mNOX-E36)	CCl_4_-liver injury mice: 20 mg/kg	Inhibition of hepatic macrophage infiltration and reduction in steatosis development. Reduced angiogenic vessel sprouting in portal vein system and fibrosis-associated angiogenesis.	[184,185]
	Blocking circulating monocyte recruitment (CCR2 antagonist RDC018)	In vivo (orthotopic mouse model of HCC and subcutaneous tumor): 30 mg/kg/day	Suppressed liver tumor growth and postsurgical recurrence. Reduced recruitment of inflammatory monocytes and TAMs. Depletion of the crosstalk between tumor cells and macrophages and suppressed M2 macrophage polarization.	[188]
	Disruption of CXCL12/CXCR4 axis (CXCR4 antagonist AMD3100)	In vitro (HCC cells, migration assay): 5 μM	Impaired in vitro migration and invasion.	[189]
	Disruption of CXCL12/CXCR4 axis (CXCR4 antagonist BPRCX807)	In vitro (HCC cells, wound healing assay): 10 µM In vivo (orthotopic mouse model of HCC): 15 mg/kg/day	Inhibition of HCC cell migration and metastatic progression. Reprogramming of TME towards antitumor immune response (M1 immunostimulatory macrophages).	[190]
Immuno- suppression	Antitumor immune surveillance (WSX1 signaling pathway)	WSX1 ^−/−^ spontaneous oncogenesis mouse model (NRAS/AKT oncogenes injection)	Tumor suppression by downregulating PD-L1 expression in tumor cells and decreasing PD-L1/PD-1 axis-induced T-cell exhaustion in tumor cells.	[191]
	Antitumor immune surveillance (CSF-1R inhibitor PLX3397)	Orthotopic mouse model of HCC: 40 mg/kg	Suppressed infiltration of TAMs, reversed M2 polarization, and decreased PD-L1 expression in HCC.	[192]

Abbreviations: CCL2, chemokine (C-C motif) ligand 2; CCR2, C-C chemokine receptor type 2; CSF-1R; colony stimulating factor 1 receptor; CXCL12, C-X-C Motif Chemokine Ligand 12; CXCR4, C-X-C Motif Chemokine Receptor 4; HCC, hepatocellular carcinoma; NASH, non-alcoholic steatohepatitis; NLRP3, NLR family pyrin domain containing 3; RIPK1, receptor-interacting protein kinase 1; TAMs, tumor-associated macrophages; TME, tumor microenvironment; TNFα, tumor necrosis factor alpha.

## 5. Outlook for the Future

Inflammation and aberrant cell death are key factors in the progression of chronic liver disease leading to HCC. Given the complexity of the signaling pathways that regulate cell death, further studies are still needed to clarify the conflicting results obtained from experiments based on the suppression of pivotal molecules such as RIPK-1 or caspase-8. Targeting cell death may be a beneficial therapeutic strategy depending on the stage of liver disease. For example, cell death inhibitors could prevent early HCC development by limiting cell damage and inflammation, thus minimizing the impact of a protumor microenvironment. Nevertheless, antiapoptotic strategies may not be effective for advanced HCC, in which the inhibition of cell death would also enhance tumor growth. In this case, harnessing the cytotoxic potential of proapoptotic/necroptotic pathways in combination with current chemotherapy and/or immunomodulatory drugs might also be a potentially interesting therapeutic approach to improving the sensitivity of patients to HCC treatment.

It is well known that a proinflammatory liver microenvironment promotes a premetastatic niche. The tumor and surrounding areas are enriched in nonmalignant cells such as CAFs, TAMs, and TAEs, which promote inflammation, angiogenesis, and fibrosis progression. Similarly, macrophage polarization contributes to HSC activation and LSEC capillarization. Progressive ECM deposition and the loss of fenestration limit the supply of oxygen and nutrients, thus triggering hepatocyte apoptosis and, subsequently, chronic inflammation. In addition, there is a dynamic interaction between nonparenchymal and tumor cells that may alter the behavior and aggressiveness of liver tumors. Therefore, a better understanding of the crosstalk between stromal components and cancer cells, and the different signaling pathways involved, is required to draw the big picture of the overall carcinogenic process in the liver.
